# Spaces of mathematical chemistry

**DOI:** 10.1007/s12064-024-00425-4

**Published:** 2024-09-11

**Authors:** Guillermo Restrepo

**Affiliations:** 1 Max Planck Institute for Mathematics in the Sciences, Inselstr. 22, Leipzig, 04103 Saxony Germany; 2 Interdisciplinary Center for Bioinformatics, Universität Leipzig, Härtelstr. 16-18, Leipzig, 04107 Saxony Germany; 3 School of Applied Sciences and Engineering, EAFIT University, Carrera 49 No 7 Sur-50, Medellin, 050022 Antioquia Colombia

**Keywords:** Chemical space, Space of reaction conditions, Space of reaction grammars, Space of substance properties, Space of substance representations

## Abstract

In an effort to expand the domain of mathematical chemistry and inspire research beyond the realms of graph theory and quantum chemistry, we explore five mathematical chemistry spaces and their interconnectedness. These spaces comprise the chemical space, which encompasses substances and reactions; the space of reaction conditions, spanning the physical and chemical aspects involved in chemical reactions; the space of reaction grammars, which encapsulates the rules for creating and breaking chemical bonds; the space of substance properties, covering all documented measurements regarding substances; and the space of substance representations, composed of the various ontologies for characterising substances.

## Introduction

Restrepo and Willett have shown that the two leading journals of mathematical chemistry, namely *MATCH Communications in Mathematical and in Computer Chemistry* (MATCH) and the *Journal of Mathematical Chemistry* (JMC), have specialised, the former, on approaches from graph theory to chemistry (Restrepo and Willett 2017a) and the latter on the mathematics of quantum chemistry (Restrepo and Willett 2017b). The aim of this document is to show that beyond graph theory and quantum chemistry, there are other worthwhile mathematical aspects of chemistry, which are presented here via five mathematical spaces.

**Fig. 1:**
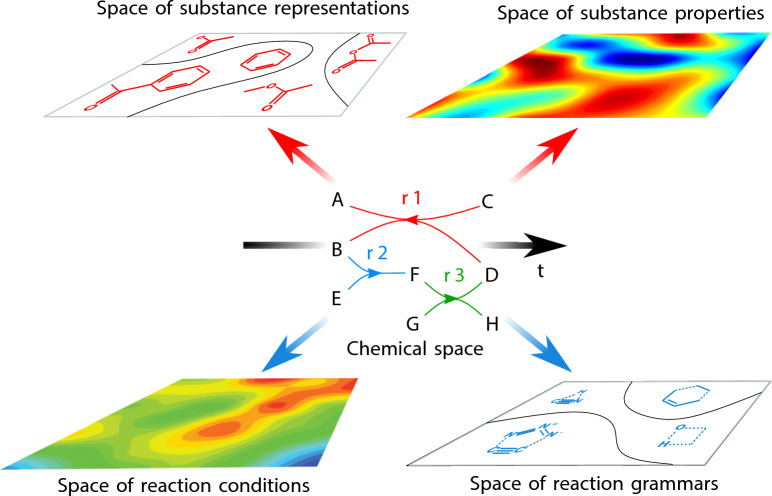
Five spaces of mathematical chemistry. The central role of the chemical space is highlighted, as well as its temporal dimension, which is extended to the other four spaces. In the chemical space, letters represent chemical substances and arrows chemical reactions (each arrow colour represents a reaction $r_i$ modelled as a directed hypergraph). In the space of reaction grammars only reaction centres are depicted for the sake of simplicity. Contour plots depicting the space of substance properties and of reaction conditions schematically indicate regions of those spaces, where the frequency of their elements is high (reddish regions) and those, where is low (blueish regions)

A graphical depiction of the spaces of chemistry is shown in Fig. 1. They correspond to mathematical spaces as they are constituted by sets endowed with a notion of nearness (Jost 2015). These spaces are: the *chemical space*, corresponding to substances and chemical reactions; the *space of reaction conditions*, spanning all pressures, temperatures, catalysts, solvents, pHs and other parameters driving chemical reactions; the *space of reaction grammars*, corresponding to the encoding of the reaction centres and their matching with educts and products for chemical reactions; the *space of substance properties*, containing chemical, physical, biological and ecological properties of substances; and the *space of substance representations*, which range from molecular graphs to geometrical structures and quantum chemical descriptions.

## Five spaces of mathematical chemistry

This section presents the five spaces of mathematical chemistry. Likewise, it provides information on what has been achieved in their study and poses new areas of research for each space and for its intertwining with the other spaces.

### Chemical space

Chemistry is all about substances and reactions, therefore the chemical space occupies a central stage in the description of the other spaces (Fig. 1). Although there are different definitions of the “chemical space,” all of them share the essence of a structure made of chemical species and of a relation among them. Thus, there are for instance definitions of the chemical space based on species that are molecular structures characterised as graphs, whose relationship is given by the nearness of their graphs, which entails all works on molecular similarity (Johnson et al. 1990; Wang et al. 2022). Other definitions of chemical space look for a geometrical similarity based on the three dimensional resemblance of the arrangement of atoms in solids (Pettifor 1984; Glawe et al. 2016). There are other approaches to the chemical space that characterise molecules in terms of their quantum chemistry descriptions, often electronic densities. In this case the notion of nearness is given by the resemblance of those quantum chemical descriptions (Carbó-Dorca et al. 2013; Carbó-Dorca 2023). A more recent account of the chemical space, although the most traditional in the history of chemistry, is that of a set of chemicals related by chemical reactions (Restrepo 2022; Llanos et al. 2019).

The question that arises is how much has been accomplished in the characterisation of those different flavours of the chemical spaces. Answering this question triggers questions on the number of substances constituting the chemical space. After all, the chemical spaces just described have the commonality of referring to substances for which it is possible to talk about their nearness.

#### Chemicals, reactions and a model for the chemical space

In this section we discuss some approaches to determine the number of possible chemicals and of reactions. In the first case the notion of a chemical as the result of atoms holding a relationship is central, which is generalised to the possible number of sets of related atoms. In the second case, determining the theoretical number of reactions given a certain number of atoms is determined by assuming a mathematical model for chemical reaction.

***Number of chemicals***

As recently discussed (Restrepo 2022), the possible number of chemicals in the chemical space depends on the number of atoms in the universe. The estimated number of particles in the universe is about $10^{80}$ (Whittaker 1945; Guggenheimer 1962; Vopson 2021),^1^ which amounts to $7\times 10^{76}$ atoms.^2^ As discussed in Restrepo (2022), a first approach to estimating the possible number of substances is determining the theoretical number of collections of atoms held together by chemical bonds. The number of such possible quasi-molecular species is given by ${\mathcal {C}}= \sum _{k=1}^{10^{76}} {k+10^{76}-1 \atopwithdelims ()k}$, where $k+10^{76}-1 \atopwithdelims ()k$ corresponds to the number of ways of selecting *k* atoms from a collection of $10^{76}$ atoms, such that order is not important and repetitions are allowed (Benjamin and Quinn 2003). This means that we are counting mono-, di, tri-,..., *n*-atomic quasi-molecular ensembles up to the ultimate largest compound made of all $10^{76}$ atoms in the universe.^3^ As having packages of atoms is not enough to have actual chemical substances, energetic conditions turn central to determine whether an atomic ensemble is chemically feasible or not. This requires determining the suitable conditions of pressure and temperature holding together the given atoms by electrostatic interactions. Although chemicals have been traditionally observed and treated at ambient conditions, there is uncharted land at extreme conditions of pressure and temperature (Yoo 2020). It is important to note that in chemistry, as well as in mathematical chemistry, we are not interested in the simultaneous “existence” of the possible substances, but rather on their theoretical possibilities.

A further piece of information that needs to be added to the possible quasi-molecular ensembles entails the diverse structural arrangements these ensembles can adopt. To approximate this, we can take a basic approach by multiplying each ensemble by the potential number of graph-theoretical representations. Since graphs are based on binary relationships between objects (in this case, atoms), these structures prove to be a suitable representation for ensembles formed by bonds connecting two atoms. Nonetheless, when we encounter substances like boranes, which do not consistently adhere to the traditional 2-centre 2-electron bonding model, a more inclusive framework is required. This broader context is provided by hypergraphs.^4^ In the context of hypergraphs, an example of a 3-centre 2-electron bond, such as the one seen in B-H-B, is represented as the hyperedge involving three atoms: {B, H, B}.^5^ Moreover, aromatic systems also translate into hyperedges, with equivalent aromatic atoms forming part of these hyperedges (Restrepo and Jost 2022). Consequently, a more precise estimation of the number of substances can be achieved by multiplying each quasi-molecular ensemble by a restricted set of potential hypergraphs associated with that specific ensemble (Restrepo 2022).

At any rate, a higher order approximation to the upper bound of the number of substances requires chemical and mathematical knowledge, and, importantly, the mathematical knowledge need not be restricted to the realm of graphs. As shown, it requires an extension to higher-order structures such as hypergraphs.^6^ Therefore, traditional collaborations between chemists and mathematicians, for instance those leading to the MOLGEN package (Gugisch et al. 2014; Kerber 2018), require further extension.^7^

When the above lines are presented to chemists, they often regard such approaches as mere mathematical diversion, far from touching chemistry reality. Moving in this direction, in the 1990s Weininger hypothesised that the number of possible organic substances under ambient conditions is about $10^{200}$, which is known as the “Weininger number” ${\mathcal {W}}$ (Warr 2022; Gorse 2006). According to Gorse, ${\mathcal {W}}$ is “a lower limit of the number of different (chiral) molecular graphs possible given known chemistry (i.e., bond types), restricted elements (C, N, O, P, S, halogens) and a molecular weight of less than 1000 dalton. Of these, it was further estimated that only about 1 in $10^{20}$ compounds could possibly be physically and chemically stable, giving $10^{180}$ compounds” (Gorse 2006). Although ${\mathcal {W}}<<{\mathcal {C}}$, ${\mathcal {W}}$ is anyhow huge.

Given the high number of possible chemicals, all flavours of chemical spaces discussed above and in further sections of this document lead to the conclusion that all chemical spaces have been very little explored (Restrepo 2022; Garcia-Chung et al. 2024).

***Directed hypergraphs as a model for chemical reactions***

Chemical space was initially modelled using directed graphs (Fialkowski et al. 2005), where, for instance a space made of the reaction A + B $\rightarrow$ C + D was modelled as the directed graph whose arcs or directed edges corresponded to A $\rightarrow$ C, A $\rightarrow$ D, B $\rightarrow$ C and B $\rightarrow$ D. The problem of this representation, as discussed in Restrepo (2022) and in Garcia-Chung et al. (2024), is that it introduces artefacts in the description that hinder the recovery of the original reaction. Thus, the sequence of four arcs just described may lead to the following set of reactions: A $\rightarrow$ C, A $\rightarrow$ D, B $\rightarrow$ C, B $\rightarrow$ D, A + B $\rightarrow$ C, A + B $\rightarrow$ D, A $\rightarrow$ C + D, B $\rightarrow$ C + D and A + B $\rightarrow$ C + D. Clearly, only the last reaction is the one provided by the actual space, but the graph representation provides by far many more options not actually provided by the chemical space.

The above shortcoming is solved by using directed hypergraphs that encode the essential feature of chemical reactions, namely that they relate sets of substances rather than individual substances (Restrepo 2022). A chemical reaction is the binary relation between sets of substances, namely the directed binary relationship between the set of educts and of products. The advantage of directed hypergraphs becomes apparent when modelling of A + B $\rightarrow$ C + D, which is now represented as the *directed hyperedge* {A, B} $\rightarrow$ {C, D}, leaving no room for alternative interpretations except for the fact that A and B react to yield C and D.^8^ The directed hypergraph representation can also be framed in a graph-theoretical version as a bipartite directed hypergraph. In this case, there are two kinds of vertices, namely substances and reactions, which are related via binary relations. For the above example, the directed bipartite graph is given by the following set of arcs: A $\rightarrow$ *r*, B $\rightarrow$ *r*, *r* $\rightarrow$ C and *r* $\rightarrow$ D. In this case *r* corresponds to the vertex representing the chemical reaction.

The hypergraph description of chemical reactions opens a new field of research in mathematical chemistry, as the mathematics of these structures is still to be developed. As discussed in Leal et al. (2021) and in Garcia-Chung et al. (2024), only a few mathematical properties of these structures have been studied, for example vertex and hyperedge degrees (Garcia-Chung et al. 2024), clustering coefficients (Estrada and Rodríguez-Velázquez 2006; Klamt et al. 2009), spectral properties (Zhou et al. 2006), curvatures (Leal et al. 2021) and more recently the Erdős-Rényi model for the random hypergraph (Garcia-Chung et al. 2024). Nevertheless, other aspects, including different random models, measures of assortativity, and betweenness centrality, among others, remain unexplored, as well as further curvatures and network geometry notions as those pioneered by Jost and collaborators (Jost and Mulas 2019; Eidi and Jost 2019; Eidi et al. 2020; Mulas et al. 2020; Leal et al. 2021; Mulas et al. 2022). On top of this mathematics to develop, the connection with chemistry is central, that is the interpretation and implications of those mathematical properties for the study of the chemical space.

***Number of reactions spanning the chemical space***

Equipped with the directed hypergraph model, we can now turn to solve the question about the possible number of chemical reactions given a certain number *n* of chemicals. As shown in Restrepo (2022), this corresponds to $3^n-2^{n+1}+1$ reactions (directed hyperedges). This is an important result allowing for the calculation of densities of chemical spaces, as it provides the background to contrast the actual number of reactions realised in a chemical space. Hence, the density of a chemical space housing *n* substances is given by $d(n)=|R|/(3^n-2^{n+1}+1)$, with |*R*| being the actual number of reactions spanning the given chemical space. The just discussed upper bound for the number of reactions also allows for devising random models for the chemical space, as recently reported when developing the Erdős-Rényi model for chemical spaces (Garcia-Chung et al. 2024).^9^ But, as discussed above, several other mathematical properties of directed hypergraphs are to be explored, and the determination of the upper bound of the number of reactions constitutes a step forward in the mathematical formalisation of the properties of directed hypergraphs.

#### Accomplishments and further open questions in the study of the chemical space

By exploring the records of reports of substances and reactions reported in the scientific literature since 1800 up to date, it has been found that the chemical space grows at an exponential rate (Llanos et al. 2019). In fact, the number of new substances doubles every 16 years and there are evidences that the number of reactions follows a similar growth pattern.^10^ Such a stable growth pattern has been possible thanks to the reliance of chemists in chemical synthesis, where they often combine no more than three educts in their reactions (Llanos et al. 2019). Of those educts, very often one is a chemical whose chemistry has been well explored, while the other chemicals are new substances in the chemical space (Llanos et al. 2019). This approach to expand the chemical space has been coined as the *fixed substrate approach*, where the fixed part is given by the chemical whose chemistry is well understood (Llanos et al. 2019). Further investigation has shown that the set of fixed chemicals is very small and only a handful of compounds have been used as anchor points to trigger new reactions leading to new chemicals (Llanos et al. 2019). These often recurring chemicals have been dubbed *toolkit compounds*. Typical examples of toolkit compounds include acetic anhydride and methyl iodide (Llanos et al. 2019).

Although the chemical space was dominated by inorganic compounds at the dawn of the 19th century, after the first quarter of that century organic chemistry began to take the lead in the expansion of the space, to the extent that today most of the chemical space is spanned by substances resulting from the combinatorial capacities of carbon, hydrogen, oxygen and nitrogen (Llanos et al. 2019).

Open questions in the study of the chemical space include determining the underlying reasons of the exponential growth of the space. Is it the result of the growth of the chemical community? Is it related to the mechanisms chemists use to connect chemicals through chemical reactions? Early ideas on the underlying mechanism of growth of the chemical space were reported by Schummer (1997). Today the readily available information on the substances and reactions reported over the history of chemistry, as well as the bibliographic information associated with chemical publications, allow for a data-driven analysis of the information, which when combined with computational and mathematical tools may shed light on the mechanisms expanding the chemical space. From a more mathematical perspective, the statistics of the directed hypergraphs are anticipated to provide insight into the geometric and topological characteristics of the chemical space and its evolution throughout the history of chemistry. Are there geometric or topological patterns in the historical expansion of the chemical space allowing for estimating the future of the chemical space?

### Space of reaction conditions

Chemical reactions reported by chemists often occur in controlled experimental settings. These settings refer to the contextual conditions of pressure, temperature, pH, reaction time, nature of the atmosphere in which the reaction mixture is embedded, as well as to the kinds of catalysts and solvents used. Today we should also include as part of the reaction conditions the nature and size of the reaction vessel. There is a strong push to move in the direction of flow chemistry (Plutschack et al. 2017), where microreactors are often used and where the “flow” properties of the substances involved are central for the outcome of the reactions.^11^

Interestingly, reaction conditions, key for affording experimental reproducibility, are often disregarded when analysing molecular structures, substances properties or the structure of the network of chemical reactions. Chemists often assume ambient conditions of experimentation; these are actually the most used reaction conditions to perform chemical reactions (Keserü et al. 2014; Jia et al. 2019). However, chemistry at extreme conditions is pushing chemists to incorporate reaction conditions in their analyses. For instance, it is difficult to understand $\text {Na}_2$He, $\text {Na}_3$Cl or $\text {NaCl}_7$ without considering the over 100 GPa required to observe these chemicals (Zhang et al. 2013; Dong et al. 2017). Likewise, it is difficult to imagine superconductivity without thinking about dropping the temperature.

As discussed before, the notion of space results central for mathematical chemistry and reaction conditions are not far from a space description. In fact, there is a *space of reaction conditions* made by the set of reaction parameters describing the reaction context, which are endowed with a nearness notion given by similarity among those parameters. Thus, similarity among real valued parameters is defined by the embedding of those parameters in a metric space, which allows, for instance, for measuring similarities among temperatures, pressures, pHs and reaction times. For chemical contextual parameters such as solvents, catalysts and atmospheric embeddings of the reaction, methods of chemical similarity such as those based on descriptor of the molecular graph or on physical properties of the substances can be applied to assess the nearness of these reaction parameters.

Reaction conditions are central for the chemical practise, as they often determine the final fate of a chemical transformation and are determining factors when competing reaction paths exist. It was just recently reported that a classic combination of amines and carboxylic acids not only leads to amides, as traditionally accepted, but to substituted amines, esters, alkanes, ketones and other families of compounds, under particular reaction conditions (Mahjour et al. 2020).

Now, that the space of reaction conditions is aimed to be expanded and further explored, it is central to understand how chemists have explored it over history and how large and diverse it is. This space becomes an important subject of research for mathematical chemistry as it offers opportunities for its mathematical characterisation. The analysis of this space could also bring new mathematics into chemistry, for instance by regarding the space of reaction conditions as a point cloud allowing for a treatment for the perspective of topological data analysis (Joharinad and Jost 2023).

### Space of reaction grammars

The expanding chemical space has brought up the need of systematising chemical knowledge at different levels, for instance by classifying chemical reactions (Schummer 1998; Restrepo and Jost 2022), or by producing abstract structures such as periodic systems encoding the salient features of chemical knowledge (Leal and Restrepo 2019). In terms of the network of chemical reactions spanning the chemical space, classifying reactions entails reducing the size and complexity of the network to an associated network of classes of substances (Bernal et al. 2015). Given the central role of reaction classification for chemical knowledge (Schummer 1998; Restrepo and Jost 2022), several approaches have been devised to meet this end, which range from the so-called “name reactions” such as Claisen condensation or Grignard reaction to more sophisticated settings involving physicochemical properties of educts and products or reaction grammars.

Name reactions rely on a manual indexing method based on the name of the chemist introducing the reaction, or on the identity of a reaction product, or on a relevant functional group involved in the transformation, or on the reagent used. At any rate, name reactions are far from being systematic (Kraut et al. 2013), which poses not only a nomenclature problem for chemistry, but makes it difficult to process the deluge of new reactions appearing every day. Physicochemical classifications are based on the resemblance of physicochemical properties of educts and products (Kraut et al. 2013). The shortcoming of this approach is its lack of a set of physicochemical properties acting as the basis for the classification, which leads to subjectivities. The approach using reaction grammars regards a chemical reaction at its atomic level, by considering educts and products as molecular structures related through a rewrite rule acting upon the structures of educts and leading to the structure of products. It is considered a grammar as it contains the information, the rule, of which bonds to break and which ones to form. Stadler and his team have pioneered this approach (Andersen et al. 2016, 2017) by computationally encoding the grammars of certain reactions and by letting them to iteratively act upon an initial set of molecular graphs.

**Fig. 2:**
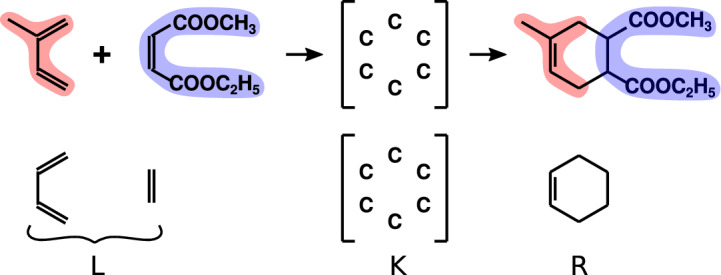
Chemical reaction grammar. Top: Diels-Alder chemical reaction with educts on the left and product on the right. Coloured regions indicate the atom mapping and the structure in brackets corresponds to the context of the reaction, that is the atoms actually involved in the reaction. Bottom: Grammar rule for the Diels-Alder reaction. L constitutes the graph of educts, K the context and R the graph of products

Figure 2 explains the concept of reaction grammar. Given a chemical reaction, as the Diels-Alder reaction shown in Fig. 2, an atom mapping algorithm detects which atoms of the educts correspond to those of the products (coloured regions in Fig. 2). This allows for detecting the context of the reactions, that is those atoms and bonds actually taking part in the reaction (Fig. 2). In the example, as all bonds around the six atoms in brackets change (four of the diene and two of the dienophile), the context is given only by these carbon atoms. The context of any reaction encodes the essential information of the transformation and therefore can be used to classify reactions in such a manner that all those reactions with common context belong in the same reaction class.^12^ The context is also called the reaction centre.

In this setting, every reaction class, such as the Diels-Alder reaction, is expressed as a triple made of the left hand side graph (L), the context (K) and the right-hand side graph (R) (Fig. 2). The former corresponds to the atoms (vertices) and bonds (edges) of the educts affected by the reaction. The context gathers together the atoms and bonds making it part of the reaction centre. In turn, the right-hand side corresponds to the atoms and bonds affected during the rewriting process. Based on this encoding of a reaction class, whenever a set of educts (molecular graphs) matches with the left hand side graph, the reaction context can be applied and the product is estimated by the rewriting rule encoded by the reaction grammar. This approach has been useful to estimate potential chemical spaces that could be obtained by the repetitive use of particular reaction classes (reaction grammars). Research in this direction is central for understanding questions, for instance, on the origin of life, where a few reaction grammars act upon a limited set of substances (Beck et al. 2004).

Equipped with the notion of a reaction grammar, then a *space of reaction grammars* can be defined as the collection of reaction grammars, whose nearness is given by their similarity. Such a similarity may be assessed in different forms, for instance by looking for the Maximal Common Subgraph (MCS) of the context of two grammars, or by including the MCS of the left hand side of the two contrasted grammars, as well as the MCS of the right-hand side graphs.

Key for characterising reaction grammars is finding the atom-to-atom mapping of educts into products. Different mathematical and computational approaches to address the detection of atom mappings have been reported over the years and today the most versatile ones include the use of Large Language Models (LLM) (Schwaller et al. 2021). In a recent study by Grzybowski it was found that despite the exponential growth of the chemical space, chemists discover new reaction centres, that is reaction classes, at a linear or sublinear pace (Szymkuć et al. 2021). This implies that despite the rapid growth of chemistry, materialised in its new substances and reactions, the innovative creation of new reaction classes is not that rapid. From a philosophical perspective, chemistry seems to be producing, in a rapid manner, new chemicals and reactions but through the same preparative methods, which makes it ponder on the actual growth of chemical knowledge (Jost and Restrepo 2023). According to Grzybowski’s results, the most used reactions by chemists to expand the chemical space are:

1. Amide synthesis from carboxylic acid and amine.
2. Alkylation of alcohols or phenols with primary or secondary halides/ O-sulfonyls.
3. Hydrolysis of esters (carboxylic acid as the main product).
4. Acylation of amines.
5. Reduction of carbonyl to alcohols.
6. Esterification.
7. Alkylation of amines with primary or secondary halides/ O-sulfonyls.
8. Oxidation of alcohols to aldehydes/ketones.
9. Acylation of alcohols/phenols.
10. Buchwald-Hartwig coupling/nucleophilic aromatic substitution with amines.

Some open questions regarding the space of reaction grammars involve, for instance, determining the most meaningful context to characterise reaction classes. As shown by Grzyboswki and collaborators (Szymkuć et al. 2021), the context of a reaction does not always distinguish different classes of reactions, and they exemplified it with the case of N-acylations and of substitutions of vinyl chlorides via addition-eliminations. In both cases, the context involves the fragments C-Cl and $\text {NH}_{2}$, but clearly both reactions cannot be classified as the same reaction. A further point, related to the space of reaction conditions, is that the fact of finding a match between the graph of educts and the left hand side of a grammar does not necessarily lead to a particular set of reaction products. As seeing in the case of the mixture of amines and carboxylic acids (Mahjour et al. 2020), shown in Sect. 2.2, the selection of the grammar finally leading to amides, or substituted amines, or esters, or alkanes, or ketones, or other families of compounds is strongly attached to the reaction conditions.

### Space of substance properties

This space is made of reported properties such as melting and boiling points, densities and biological activities, among several others (Schummer 1998), whose nearness is quantified by the distance between actual property values. These distances result from embedding the properties in a metric space.

An early attempt to analyse the behaviour of a single property was carried out by Grzybowski and his team (Fialkowski et al. 2005), who studied the molecular weight of all organic substances reported between 1850 and 2004. It was found that chemists use raw materials of about 150 g/mol to reach products weighing, on average, 100 g/mol more. Other studies have focused on the 21st century behaviour of the subspace of pharmacological substances, with emphasis on their molecular weight and logarithm of the octanol/water partition coefficient (Paolini et al. 2006). These properties are suitable proxies for the oral-drug like activity of those compounds (Hann 2011).

Despite these tailored studies of the space of properties, there is no systematic study of the substance properties reported by chemists. Therefore, analysing the space of substance properties constitutes an interesting field of research for mathematical chemistry. And the moment is ripe to begin these studies as the large volume of chemical records today stored in electronic databases offers ample possibilities to analyse the size, diversity and temporal expansion of the properties reported by chemists about the substances they have procured either by extraction or by chemical synthesis.

Here, once more, topological data analysis (TDA), as shown in Sect. 2.2, becomes central. As the space of substance properties can be modelled as a point cloud upon which simplicial complexes are defined, the insights of TDA are expected. By using TDA, the notion of connectivity in this space can be formally addressed and it opens the possibility of studying tunnels, voids and higher-dimensional structures inherent to substance properties. This information may provide structural proxies of the space of substance properties, whose dynamics can be further explored by analysing the space on an annual basis and over history.

### Space of substance representations

There are different ontological levels for chemistry, which range from bulk substances to quasi-molecular species^13^ (Restrepo and Harré 2015). Chemical specialisation, with the emergence of subdisciplines, has led to substance representations proper of each subdiscipline. This is clearly seen, for instance, in the amino acid sequence of interest for biochemists, where often the internal molecular structure of amino acids is irrelevant. Or in solid state chemistry, where geometrical structures of atoms are central and, where often the topological sequence of atoms or clusters of them play a small role. Chemical annotation and nomenclature, over history, have spanned all these ontological levels of chemistry. The subject of the ontology of chemistry is also a computational challenge, where mathematics can also play a major role. Current discussions on groups pushing for open science, open data and code in chemistry are discussing about the appropriate computational ontology for chemistry, able to encompass all its different subdisciplines.^14^

Before the acceptance of molecular structural theory, substances were labelled by their composition, then by the binary notation following the dualistic theory by Berzelius (Jost and Restrepo 2023). Today, a large part of the ontology of chemistry revolves around molecular structures represented as labelled graphs and encoded via SMILES or InChI structures. When needed, these encodings are endowed with geometrical information, where the connectivity relationships among atoms belonging to the molecular graph are enriched by interatomic distances. This is the case of crystal structures or of substances whose solid state is of relevance. Likewise, the chemical ontology may be enriched by introducing information on the nanoscale arrangements of quasi-molecular species, which are of relevance for new materials chemistry and nanotechnology, for instance.

Therefore, the space of substance representations involves selecting the ontology in place for chemistry and the assessment of the similarity among those substance representations. For an ontology of compositions the similarity can be assessed in terms of lexicographic resemblance, but it can also be addressed by considering the role of the substances in the chemical space, as reported in Leal et al. (2022). For graph-theoretical representations, the usual similarity is based on the determination of the MCS of the graphs compared. As in the case of the composition, similarity among molecular graphs can also be assessed by considering the role of the analysed graphs in the chemical space. Initial results in this direction use LLM to analyse the similarity among chemicals (Jablonka et al. 2023).

**Fig. 3:**
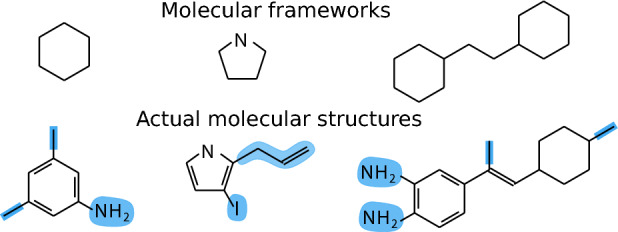
Molecular structural patterns. Most produced molecular frameworks and some actual molecular structures containing them. Blue fragments correspond to side-groups

Early results analysing the space of molecular structures only consider the period 2009–2018 and are restricted to the organic chemistry part of the whole chemical space (Lipkus et al. 2019, 2008). It is found that chemists have actually reported very few *molecular frameworks*, that is general molecular templates depicting atom connectivity and disregarding bond order (Lipkus et al. 2019, 2008) (Fig. 3). Interestingly, a large fraction of the chemical space is spanned by a reduced set of these frameworks. In Fig. 3 the three most populated frameworks are depicted. The finding that chemists do not populate the chemical space with a large diversity of molecular frameworks is presumably related to the conservatism in the selection of starting materials to undergo chemical synthesis (Llanos et al. 2019; Bishop et al. 2006), a conservatism that has been also found for the subspace of pharmaceutical chemicals (Brown and Boström 2016).

Despite these results, a wider analysis of the space of molecular structures is still to be done, where not only organic but inorganic, organometallic and biochemical substances are to be included. This poses serious challenges and opportunities for mathematical chemistry. For instance, the SMILES and InChI annotation of inorganic substances is still an open problem, as well as the annotation of large structures such as proteins and new materials.^15^ There is another aspect of the space of substance representations which has not been considered yet, namely side-groups. These are molecular fragments that remain after extracting molecular frameworks. In Fig. 3 they are highlighted in blue. Although the molecular backbone, gauged through the molecular frameworks, provides information about the building blocks of the chemical space, all those side-groups that make the production of new chemicals possible are an important part of the exploration of the chemical space.^16^

## Relationships among spaces of mathematical chemistry

Besides the chemical and mathematical richness of the five spaces mentioned in the previous sections, much more chemical and mathematical insight can be obtained by exploring their relationships. We begin by the binary relationship between spaces and then move to describe a few of their higher-order relationships. For the sake of simplicity, these relationships are presented through questions the relationships trigger.

### Chemical space—space of reaction conditions

To what extent is the chemical space affected by variations of reaction conditions? That is, to what extent the shape of the network of chemical reactions spanning the chemical space has been affected by the discovery and use of particular reaction conditions? Has the frequent synthesis of amides from carboxylic acids and amines led to an overpopulation of its associated reaction conditions? How the often production of these amides has affected the connectivity, curvature and properties of the network spanning the chemical space? From a more recent perspective, has the move towards extreme conditions or the introduction of flow chemistry left its mark on the structure of the hypergraph network? Are there reaction conditions leading to particular local structural properties of the chemical space? If we perform reactions outside the ambient conditions on Earth, what can we say about the structural properties of the chemical space? Some results in this direction involve the analysis of astrochemical networks (Solé and Munteanu 2004).

### Chemical space—space of substance representations

How do different ontologies of chemistry affect the description of the chemical space? To what extent is the chemical space invariant to changes in substance representation? What does this tell us about the underlying structure of chemistry? These questions go hand in hand with the technologies required to devise substance representations. For instance, the different crystal forms of ice cannot be attained without the ensemble of high-pressure technology and the spectroscopic apparatuses required to observe those forms (Rosu-Finsen et al. 2023). Thus, the chemical space-space of substance representations relation sheds light also on the stability or invariability of the chemical space regarding technologies.

A further avenue of research for the relationship chemical space-substance representations entails characterising substances not only by highlighting their internal structures as done when using the graph model of molecules. Molecular graphs are suited for small molecules, for which most of their chemistry is encoded in their covalent backbone that includes the presence of particular functional groups. But chemistry is much more than small molecules and covalent structures. As discussed in Sect. 2.1.1, boranes represent a challenge for graph-theoretical descriptions, which are better described by using hypergraphs. The same applies to proteins, as will be shown in Sect. 3.6, whose features are often the result of weak interactions not encoded in their covalent graph-theoretical backbone and which could be better described by using higher-order relationships such as hypergraphs. Thus, substance representation can be enlarged by including hypergraph characterisations.

Graph and hypergraph representations of substances are, nonetheless, intrinsic characterisations of the atoms holding together chemical species. A further and appealing substance characterisation is provided by the chemical space. In this setting, the emphasis is not intrinsic but rather extrinsic, as substances are characterised by their connectivity in the chemical space, that is in the network of chemical reactions. A substance, in this setting, is not characterised by what it contains but rather by what it produces and what it is produced. Thus, a challenge for the chemical space-space of substance representations relationship lies in exploring the identity of chemicals in terms of their chemical relationships.

### Chemical space—space of substance properties

The just described network-like representation of substances opens the door to new questions. For instance, how QSAR^17^ methods could be developed that operate at the network-like level of representation of substances, instead of at the traditional level based on molecular structures? That is, instead of claiming that the boiling point of alkanes can be modelled using graph-theoretical properties, such as the Wiener index of alkanes’ molecular graphs (Wiener 1947), can we say that a particular property of a family of chemicals can be modelled via the curvature of those chemicals, calculated based on their local connectivity in the chemical network spanning the chemical space? Some theoretical developments in this direction were early reported using basic notions of category theory (Bernal et al. 2015). Nevertheless, much more research in this subject is required.

### Chemical space—space of reaction grammars

How different would be the chemical space if the grammars of chemistry were different? This question connects the discussion on the spaces of mathematical chemistry with the field of artificial chemistries (Fenizio 2011).^18^ We can perfectly imagine grammars outside those already known in chemistry and let them evolve by computational modelling. Is the structure of the resulting chemical space rather different from the structure we know for the current chemical space? Questions of this sort are addressed in studies on the origin of life (Ono et al. 2008), where given a set of chemicals and of available grammars, the system is allowed to evolve by the action of those grammars. Central grammars in these discussions involve those allowing for autocatalytic reactions (Semenov et al. 2016).

The relation chemical space-space of reaction grammars has a further avenue of research by incorporating human agency in the composition or connection of grammars. That is, whereas the formose reaction leads to a gigantic chemical space when initially acting upon formaldehyde (Andersen et al. 2013), most of the reactions used in wet-lab chemistry (Szymkuć et al. 2021) require human action in the selection of educts to start reactions and also in the separation and purification of products in batch chemical processes (Plutschack et al. 2017). A more realistic account of the relationship chemical space-space of reaction grammars should involve the description of the human action as a new kind of grammar able to connect existing chemical grammars.^19^

### Space of reaction grammars—space of reaction conditions

What is the region in the space of reaction conditions associated with each grammar? This leads to wonder whether there are regions in the space of reaction conditions that are only associated with a particular grammar. In Sect. 2.2 it was shown how variations on reaction conditions led to different products in an original mixture of carboxylic acids and amines. This illustrates how alterations in reaction conditions, often linked to a particular grammar, can result in the formation of different grammars. This example highlights the new areas that can be investigated by examining the connection between grammars and reaction conditions, where topological concepts like continuity become crucial for deepening our understanding of this relationship.

The grammar-reaction condition relationship may trigger research on innovation in chemistry. It is known that most of the chemistry already reported has been conducted in a narrow region of the space of reaction conditions (Keserü et al. 2014). To what extent moving outside that region would lead us to new territory in the space of grammars? Are extreme conditions and flow chemistries leading us to new reaction grammars?

### Space of reaction grammars—space of substance representations

To what extent new substance representations, as those shown in Sect. 3.2, that are based on a network-like description of chemicals, can be instrumental in the discovery of new reaction grammars? Can we, instead of looking for graph matchings between left hand side parts of grammars and molecular graphs of reaction educts (Fig. 2), look for hypergraph matchings and elaborate all the theoretical construct of reaction grammars based on those higher-order matchings?

The above description was based on a network-like account of chemical substances. In Sect. 2.1.1 we discussed an alternative approach to substance representation, based on hypergraphs rather than on graphs. Can we develop new grammar frameworks based on that internal hypergraph representation of substances? Research in this direction would lead to study hypergraph isomorphisms, which is a vibrant field of mathematics (Feng et al. 2023). Moreover, this research could unveil hidden relationships between substances and their reactivity (this latter is encoded in reaction grammars). It is well known that substance reactivity is not always encoded in its covalent backbone, as is the case of proteins. Very often the reactivity of these compounds is encoded in higher-order interactions, as is the case of the chemistry encoded in H-bonds and weak interactions (Sukenik et al. 2017). In this setting a more appropriate level of protein description would be a hypergraph model. In fact, this justifies the current level of description of proteins, as shown in Sect. 2.5, which is based on sequences of amino acids, rather than on gigantic covalent molecular structures.

### Space of reaction grammars—space of substance properties

How substance properties determine the kind of grammars in which substances participate? This is the question running across the history of theoretical chemistry, from dualistic and acid–base theories to theories relying on electronic density. That is why we cannot imagine a neutralisation reaction without thinking of acids and bases, for instance.

Given a reaction grammar, artificial or not, can we infer, based only on the grammar, the required substance properties to trigger the action of the grammar?

The grammar-substance property relation is also central for the search of life beyond Earth. Are there particular substance properties leading to autocatalytic grammars or to central grammars to sustain life? This is the path followed by several researchers analysing the possibility of having life in other corners of the universe. In these researches, often the focus is on a particular substance properties, for instance the IR spectrum of a planet, which sheds light on the availability or not of water and carbon dioxide, which are believed essential for sustaining life (Clery 2024).

### Space of substance properties—space of substance representations

How the ontology of chemistry narrow the scope of substance properties? (Jost and Restrepo 2023). It is well known how molecular structural chemistry brought order and predictive capacity to organic chemistry (Jost and Restrepo 2023; Klein 2003). But it also provided a narrow way of thinking about substances (Jost and Restrepo 2023) as it left aside chemicals not explained under the framework of organic molecular structures. This was the case, for instance, of ferrocene, which in spite of being synthesised several times, its structure was wrongly assigned under the framework of covalent molecular structural theory (Seeman and Cantrill 2016).^20^

Overall, the framework of molecular structures left its mark on the space of substance properties as the lack of unforeseeable compounds begets a lack of substance properties, presumably of novel character, especially if we attend to the molecular structure–property principle.^21^ Famous examples include polymers, which were initially not regarded as compounds, but rather as colloidal aggregates (Mülhaupt 2004), whose discovery and further development populated the space of substance properties in a rather unexplored region, namely of high molecular weights.

A challenge to the relationship substance representation-substance properties is to generalise the accumulated QSAR knowledge, based on graph representations, to hypergraph representations. That is, instead of seeking at having a relationship of the sort $P=f(\text {molecular structure})$, we could think of $P=f(\text {hypergraph})$, where the hypergraph may refer to (i) the internal higher-order relationships among atoms in molecules or to (ii) the network-like description of a chemical in the chemical space (Sect. 2.1.1).

Results in the direction of enriching the relationship substance representation-substance properties involve the advancements in InChI annotation, which if generalised to account for the different ontological levels of chemistry, may lead to relationships of the sort $P=f(\text {InChI})$, with an ideal InChI ranging from substance composition to higher-order relationships (ChemistryViews 2024; Restrepo and Jost 2022).^22^

A case in point of how updates on substance representations are related to substance properties is the case of nanomaterials, including quantum dots. In these cases, the nanometric description of the new materials is essential for understanding the properties these materials depict (Baig et al. 2021; Jang and Jang 2023).

### Space of substance representations—space of reaction conditions

As discussed in the previous section, chemistry holds an ontological hierarchy (Restrepo and Harré 2015). These ontological levels range from composition to nano- and molecular structure. What is the relationship between these ontological levels and the reaction conditions allowing for further refinements of the ontology?

An example of how particular reaction conditions lead to new ontological species include the syntheses of fullerenes using discharge arcs, where temperatures exceed 3000 C and pressures often range between the 13 and 27 kPa under helium or argon atmospheres (Kroto et al. 1985). Fullerenes are currently becoming new “atoms” of chemistry, as they become conservative parts in chemical reactions that are transferred during chemical reactions without further chemical alteration. Key for fullerene chemistry involves the high-order structures formed by fullerene units within their compounds,^23^ including monolayers made of fullerenes (Hou et al. 2022), as well as the various structures generated by the combinatorial potential of fullerene species (Chang et al. 2024).

### Space of reaction conditions—space of substance properties

Are there regions of substance properties that cannot be attained by a particular collection of reaction conditions? This motivates the study of continuity properties between the space of reaction conditions and the space of substance properties. That is, how related are the neighbourhoods of reaction conditions with the neighbourhoods of substance properties?

A subject of interest in this reaction condition-substance property relation is the thermodynamic vs. kinetic control of reactions to attain chemical products with different properties. Typically, high temperatures or long reaction times allow the system to reach equilibrium (thermodynamic control), which favours the synthesis of the most stable products (thermodynamic product). In contrast, reactions conducted at lower temperatures or with shorter reaction times, lead to products that form faster (kinetic products) and that not necessarily are the most stable ones (Zhang and Yu 2023).

### Higher-order relationships among spaces of mathematical chemistry

In Sects. 3.1–3.10 we have addressed the binary relationships between the space of mathematical chemistry. That is, the $n \atopwithdelims ()k$ relations for the $n=5$ spaces of mathematical chemistry with *k*-ary ($k=2$) relationships. Exploring in full the *k*-ary relationships for the five spaces of mathematical chemistry would require analysing those with $k > 2$, that is $\sum _{k=3}^{5}{5 \atopwithdelims ()k}=16$ relationships, which are beyond the scope of this paper. Instead of going in that direction, we just mention some of these higher-order relationships worth exploring for chemistry and mathematical chemistry.

Research in this high-order relationships should involve devising new approaches to formalise reaction grammars that incorporate new ontologies for chemistry. In particular, those mentioned in this document, namely (i) the characterisation of substances based on network features of chemicals in the chemical space and (ii) on higher-order relationships of atoms in chemical species via hypergraphs. The characterisation of those grammars should also incorporate the role of reaction conditions, which must not only affect the application of the grammars but the ontological nature of chemicals. After all, substances’ proclivity to react or not in certain fashion is determined to a large extent by the conditions in which they are embedded. That is, the mutual relationship between substance properties and reaction conditions and its relationship with reaction grammars is worth studying, which involves characterising substances as usual via molecular graphs, but also using the higher-order structures discussed in this work. This analysis will provide a holistic account of the chemical space, which can be used to understand the dynamics of the historical expansion of chemistry and of all aspects of its material output represented in new substances, reactions, properties, reaction conditions and ontologies.

## Conclusion and outlook

We have provided the description of five spaces of relevance to trigger further research in mathematical chemistry. They are the chemical space, the space of reaction conditions, the space of reaction grammars, the space of substance properties and the space of substance representations. Besides describing them in terms of the elements that constitute them and the relationship that holds together those elements, we provided some examples of important relationships between couples of those spaces, which either have become subjects of research in mathematical chemistry or that constitute potential avenues of research. The presentation of these spaces has been nuanced by the incorporation of higher-order structures in mathematical chemistry, especially of hypergraphs. This lifting of higher-order structures aims at showing that mathematical chemistry, besides delving into the wonders of graph theory and the mathematics of quantum chemistry, may also explore new avenues of research in other mathematical structures, which may eventually contribute to bringing insight to chemistry as one of the outcomes of mathematical chemistry research.

## Footnotes

1 As reported in Restrepo (2022), early calculations were reported by Eddington in 1931 and account for the number of hydrogen atoms spanning the mass of the observable universe (Whittaker 1945). Eddington’s calculation was based on hydrogen, taking into account that about 75% of the mass of the universe is provided by this element. Although Eddington’s number ($2.36\times 10^{79}$) can be obtained by dividing the mass of the universe ($1.45\times 10^{53}$ kg) by the mass of a hydrogen atom ($1.67\times 10^{-27}$ kg), the figure has been refined to include the number of baryons and electrons in the universe, which amounts to $1.93\times 10^{80}$ particles (Vopson 2021).

2 By considering the abundances of elements in the universe (Greenwood and Earnshaw 2012; Vopson 2021) and their atomic weights, the number of atoms per element can be calculated, which leads to the total number of atoms in the universe. See the Supplementary Information in Restrepo (2022) for a complete account of the number of atoms per element in the universe.

3 As discussed in Restrepo (2022), this upper bound requires further adjustments to touch physical and chemical ground. It requires taking a few atoms out of the $10^{76}$ to account for the synthesiser of the largest compound, which may be either a human or a robot.

4 In a *hypergraph* $H=(V,E)$, *V* is a set of vertices and *E* is a collection of *hyperedges*, that is of sets of vertices of any size. So, for instance, for $V=\{a,b,c\}$ a possible hypergraph is $H=\{\{a,b,c\},\{\{a,b\},\{a,b,c\}\}\}$, as well as $H'=\{\{a,b,c\},\{\{a,b\},\{b,c\}\}\}$. Note that $H'$ also corresponds to a graph. In fact, graphs are a particular case of hypergraphs.

5 For instance, assuming we can distinguish between atoms of the same element, following the typical molecular representations, the hypergraph model for $\text {B}_{2}\text {H}_{6}$ would look like {{B, B, H, H, H, H, H, H}, {{B, H}, {B, H}, {B, H}, {B, H}, {B, H, B}, {B, H, B}}}. Similarly, the model for $\text {H}_{2}\text {O}$ would be {{H, H, O}, {{H, O}, {H, O}}} (Restrepo 2022).

6 Other further higher-order structures of potential use in mathematical chemistry include simplicial complexes (Mulas et al. 2022).

7 Among several other features, MOLGEN provides the number of isomers of a given chemical formula based on a blending of group and graph theories, along with group algebra.

8 Formally, a chemical reaction $r: E \rightarrow P$, with *E* being the set of educts and *P* the set of products, is modelled through the directed hyperedge $r=(E,P)$. In general, given a chemical space with substances gathered in *X* and reactions collected in *R*, the chemical space is modelled with the directed hypergraph $H=(X,R)$.

9 Actually, the model developed in Garcia-Chung et al. (2024) was devised for chemical hypergraphs, which are a simplification of directed hypergraphs, as the direction is disregarded. Hence, the Erdős-Rényi model, as detailed in Garcia-Chung et al. (2024), aligns with the chemical space model, in which substances involved in reactions are segregated into products and reactants, but it is impossible to discern between the two sets.

10 This arises from results by Grzybowski, who determined the growth rate of reactions and substances for the subspace of organic chemistry (Fialkowski et al. 2005).

11 Flow chemistry is also a vibrant field of further development for mathematical chemistry, at the border of mathematical physics, where fluid dynamics and turbulence theory may play an important role in providing insight and understanding of the processes occurring in the microreactors used in this kind of chemistry (Plutschack et al. 2017).

12 This approach was recently followed by Grzybowski and his team, when analysing the number of reactions classes of organic chemistry (Szymkuć et al. 2021).

13 Examples of quasi-molecular species include molecular clusters, van der Waals complexes and other chemical species (van Brakel 2012).

14 Discussions of this kind are at the basis of the different substance annotation systems in chemistry, for instance of the InChI system.

15 While reviewing this document, it was announced that the new release of InChI has addressed the issue of annotating inorganic and organometallic compounds (ChemistryViews 2024).

16 This is an aspect suggested by Stadler in a private communication (September 2021).

17 QSAR stands for Quantitative Structure-Activity Relationships, which aim at relating the molecular structure of substances with their activities. Originally, “activity” was regarded as the pharmacological effects of chemicals, but the term was latter extended to mean all sort of substance properties, from physical to chemical and ecological ones.

18 An artificial chemistry is a computational setting in which a pool of chemical representations (often using the graph-theoretical model) and a set of reactions, encoded as reaction grammars, are allowed to interact. The artificial chemistry arises by the rapid appearance and further reaction of new chemical species allowed for the grammars. We note in passing that not all chemical grammars allow for such a growth. See main text in this section.

19 The role of human action was highlighted by Jeffrey A. Johnson in January 2024 and its abstraction has been mainly carried out by Alejandro Garcia-Chung (work in progress).

20 Ferrocence and metallocenes, in general, pose a challenge to computational substance ontologies, which are best suited for small covalent organic compounds. See Sect. 2.5.

21 This principle states that substance properties are determined by the substance molecular structure (McKinney et al. 2000). This is at the core of QSAR approaches.

22 As InChI structure holds a hierarchy of ontologies, methods as those introduced by Klein could (Restrepo and Klein 2011) be used that define a partially-ordered-set structure on the hierarchy and that operate upon the ordered structure. In this case, $P=f(\text {InChI})$ is actually seen as $P=f(\text {poset(InChI)})$.

23 This does not mean that chemistry of single units of fullerene is not conducted. In this respect, see for instance the synthesis of endohedral fullerenes, where fullerene units encapsulate a metal in their interior (Popov et al. 2013).
